# Mapping Psychiatric Diagnoses in Free Text to ICD Codes Using Natural Language Processing (NLP) and Machine Learning

**DOI:** 10.1192/j.eurpsy.2026.11864

**Published:** 2026-07-31

**Authors:** H. Jimenez-Garcia, R. Lara-Cabrera, M. Salvador-Robert, A. Diaz-Alvarez, M. L. Barrigon, E. Baca-Garcia, A. De La Torre-Luque

**Affiliations:** 1 Polytechnic University of Madrid; 2 King Juan Carlos University; 3 Gregorio Marañon University Hospital; 4 Jimenez Diaz Foundation Hospital; 5 Complutense University of Madrid; 6 CIBERSAM ISCIII, Madrid, Spain

## Abstract

**Introduction:**

Mental health is a major public health concern. Over one in three people reported mental problems in 2023, and suicide is the second leading external cause of death. Manual coding of psychiatric diagnoses from free text into ICD-10 slows clinical workflows, introduces errors, and hinders research. Automating this process with natural language processing (NLP) and machine learning (ML) can improve consistency, reduce administrative burden, and assist clinicians in improving diagnostic accuracy and treatment planning.

**Objectives:**

1) to clean and normalize an anonymous corpus of 79,048 Spanish psychiatric notes; 2) to evaluate semantic representations and identify the most suitable Spanish biomedical embeddings; 3) to develop and compare multilabel classification models, including XGBoost, random forests, neural networks, hierarchical models, and large language models (LLMs) fine-tuned with QLoRA; 4) to measure performance and computational cost.

**Methods:**

The dataset from a Spanish hospital was used for model training. It contained more than 150,000 diagnostic labels referring to 83 ICD-10 codes and was preprocessed to correct errors, normalize codes, and replace abbreviations. Several embedding models were tested, with multilingual e5-large selected as the most effective. This representation was used as input to classical classifiers, neural networks, hierarchical models grouped by diagnostic families, and LLMs. Training and evaluation followed a stratified split, using F1, precision, and recall as metrics. All experiments were executed on a single NVIDIA RTX 4090 GPU.

**Results:**

The best balance between accuracy and cost was achieved with multilingual e5-large embeddings and multilabel XGBoost, reaching F1 = 0.72 with four minutes of training (Figure 1). A neural network on top of the encoder increased F1 to 0.76, at 37× higher cost. The hierarchical family-based approach lowered combined performance to F1 = 0.667 due to error propagation, with overall results ranging from 0.37 (Disorders of adult personality and behaviour) to 0.78 (Mood disorder) across models. Llama-3 LLMs fine-tuned with QLoRA improved from near-zero performance to F1 = 0.62, showing potential but still underperforming compared to embedding-based models. All approaches were computationally feasible within limited resources.

**Image 1:**
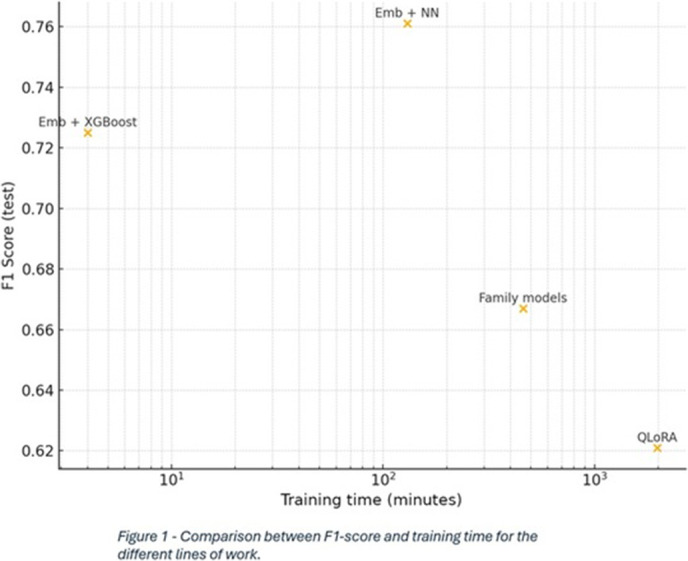

**Conclusions:**

Automatic mapping of psychiatric notes to ICD-10 codes is feasible and clinically valuable. The embedding + XGBoost pipeline offers the most practical trade-off for immediate deployment, while fine-tuned encoders provide higher accuracy when computational budgets allow. Fine-tuned LLMs represent a promising medium-term option if larger corpora and hardware are available. Limitations include short note length, class imbalance, and labelling variability. Future work will explore active learning with experts, synthetic data generation, and adaptation to ICD-11.

**Disclosure of Interest:**

None Declared

